# Incidence and risk factors of preterm birth in a rural Bangladeshi cohort

**DOI:** 10.1186/1471-2431-14-112

**Published:** 2014-04-24

**Authors:** Rashed Shah, Luke C Mullany, Gary L Darmstadt, Ishtiaq Mannan, Syed Moshfiqur Rahman, Radwanur Rahman Talukder, Jennifer A Applegate, Nazma Begum, Dipak Mitra, Shams El Arifeen, Abdullah H Baqui

**Affiliations:** 1 International Center for Maternal and Newborn Health (ICMNH), Johns Hopkins Bloomberg School of Public Health, Johns Hopkins University, 615 N. Wolfe Street, Room # E8624, Baltimore, MD 21205, USA; 2 Department of Health and Nutrition, Save the Children USA, 2000 L Street NW, Suite # 500, 20036 Washington DC, USA; 3 Family Health Program, Global Development Division, The Bill and Melinda Gates Foundation, Seattle, WA, USA; 4 Ma-Moni Project, MCHIP/Save the Children, Bangladesh Country office, Dhaka, Bangladesh; 5 International Centre for Diarrheal Disease Research, Bangladesh (icddr,b), Mohakhali, Dhaka, Bangladesh

**Keywords:** Preterm birth, Risk factors, Bangladesh, Community-based program

## Abstract

**Background:**

Globally, about 15 million neonates are born preterm and about 85% of global preterm birth occurs in Asia and Africa regions. We aimed to estimate the incidence and risk factors for preterm birth in a rural Bangladeshi cohort.

**Methods:**

Between June 2007 and September 2009, community health workers prospectively collected data from 32,126 mother-live-born baby pairs on household socio-demographic status, pregnancy history, antenatal care seeking and newborn gestational age determined by recall of date of last menstrual period.

**Results:**

Among all live births, 22.3% were delivered prior to 37 weeks of gestation (i.e. preterm); of which 12.3% were born at 35–36 weeks of gestation (late preterm), 7.1% were born at 32–34 weeks (moderate preterm), and 2.9% were born at 28–31 weeks of gestation (very preterm). Overall, the majority of preterm births (55.1%) were late preterm. Risk of preterm birth was lower among women with primary or higher level of education (RR: 0.92; 95% CI: 0.88, 0.97), women who sought antenatal care at least once during the index pregnancy (RR: 0.86; 95% CI: 0.83, 0.90), and women who had completed all birth preparedness steps (RR: 0.32; 95% CI: 0.30, 0.34). In contrast, risk of preterm birth was higher among women with a history of child death (RR: 1.05; 95% CI: 1.01, 1.10), who had mid-upper arm circumference (MUAC) ≤250 mm, indicative of under nutrition (for women having MUAC <214 mm the risk was higher; RR: 1.26; 95% CI: 1.17, 1.35), who reported an antenatal complication (RR: 1.32; 95% CI: 1.14, 1.53), and who received iron-folic acid supplementation for 2–6 months during the index pregnancy (RR: 1.33; 95% CI: 1.24, 1.44).

**Conclusions:**

In resource poor settings with high burden of preterm birth, alike Bangladesh, preterm birth risk could be reduced by close monitoring and/or frequent follow-up of women with history of child death and antenatal complications, by encouraging women to seek antenatal care from qualified providers, to adopt birth preparedness planning and to maintain good nutritional status. Additional research is needed to further explore the associations of antenatal iron supplementation and maternal nutritional status on preterm birth.

## Background

Recent global estimates suggest that more than 1 in 10 or an estimated 15 million babies born in 2010 were preterm, of which more than 1 million died as a result of preterm birth and related complications [1]. Although neonatal mortality rates have fallen globally between 1990 and 2009 [2], the absolute numbers and rates of preterm birth have increased during this period [3]. Preterm birth complications account for 35% of the estimated 3.1 million global neonatal deaths [4], and are the second leading cause of death in children under 5 years of age. The vast majority (85%) of global preterm births occur in Asia and Africa [5] where health systems are weak and access to and utilization of health services are limited, contributing to the higher risks of death and disabilities in preterm babies [6,7]. Approximately one-third of preterm survivors suffer from severe long-term neurological disabilities, such as cerebral palsy or mental retardation [8]. Furthermore, preterm infants carry increased risk of a range of neurodevelopmental impairments and disabilities, including behavioral problems, school learning difficulties, chronic lung disease, retinopathy of prematurity, hearing impairment, and lower growth attainment [9]. Preterm birth affects not only infants but also their families who may have to spend substantial time and financial resources to ensure care for their preterm infants; thus, preterm birth has increasing cost implications for families and health services [10].

Identification of at-risk women and their risk factors for preterm birth is important for targeting of services and initiation of risk-specific interventions and/or preventive measures. Study of risk factors might also provide important insights leading to new discoveries for prevention and management of preterm births. We describe the burden and associated risks factors of preterm birth in a cohort of rural Bangladeshi women.

## Methods

### Study design

We analyzed prospectively collected data from a large community-based cluster-randomized trial conducted in Sylhet district of Bangladesh. Data for our study were primarily collected to evaluate the impact of two regimens of umbilical cord cleansing - single-day vs. 7-day - with 4.0% chlorhexidine solution on all-cause neonatal mortality and incidence of cord infections [11,12].

### Study setting and population

The study was implemented during June 2007- September 2009 in 22 unions (the smallest administrative unit with a health center) in 3 rural sub-districts (called *upazila*) of Sylhet district (Beanibazar, Zakiganj and Kanaighat) in north-eastern Bangladesh with an estimated total population of 546,000 people. The study area was divided into 133 working units (clusters) each served by a female community health worker (CHW). CHWs implemented the interventions and collected data from respondent women and their babies.

### Study implementation

Details of the study designs, interventions, delivery strategies and the map of the area have been published elsewhere [11,12]. Briefly, CHWs followed a complete map of all households and thus prepared a complete list of all married women of reproductive age (MWRA) through house-to-house visitation and recorded their names, addresses and pregnancy status. The list was updated and new pregnancies were identified every two months by conducting home visits. All newly identified pregnant women were invited to participate in the study and explained the study procedures. Those agreeing to participate gave informed oral consent and provided data on age, parity, date of last menstrual period, literacy, a brief pregnancy history, and socio-economic information about the household.

All enrolled women were provided with a package of maternal and newborn health interventions, delivered by CHWs through two antenatal home visits. The first session was conducted at the time of enrolment at 12–16 weeks of pregnancy and the second occurred at approximately 32 weeks of pregnancy. The intervention package included a clean delivery kit (CDK), messages on birth and newborn care preparedness (BNCP), and advice on essential newborn care (immediate breastfeeding, thermal care and clean cord care) and on neonatal danger sign recognition and care-seeking [11,13]. At each visit, information was collected by CHWs on status of birth and neonatal care preparedness, antenatal care (ANC), complications during pregnancy and care seeking for those complications. BNCP included practice of the following 6 steps: 1) selection of birth attendant, 2) selection of newborn care personnel, 3) arrangement for three pieces of cloth for drying/wrapping of the newborn, 4) arrangement for transport for any emergency need, 5) savings for management of complications, and 6) having a CDK for use during delivery.

### Inclusion and exclusion criteria

All reported live births within the study area for which data was available on the first day of the last menstrual period (LMP) were included in this study. Reported stillbirths and abortion (spontaneous, induced or therapeutic) were excluded. Pregnancies terminated before 28 weeks of gestation were defined as miscarriage/abortion. A stillbirth was defined as an infant born without any signs of life (no spontaneous crying, breathing, and/or movement) at 28 weeks of gestation or later.

### Assessment of exposure variables

Socio-demographic and economic information (women’s age at delivery, educational attainment of women and their husbands, basic housing construction, household belongings, religion) and previous pregnancy history were collected by CHWs using a structured instrument in face-to-face interviews during the enrolment visit. Relevant data on antenatal care seeking, compliance with BNCP, consumption of supplied iron tablets, TT immunization dosage and antenatal complications (history of fever, severe abdominal pain, swelling of hand, leg or face, vaginal bleeding, convulsion, severe headache, blurring of vision) were also collected from all women during BNCP visits or the first postpartum visit. Compliance with BNCP was categorized as “fully compliant” (woman reported practice all 6 of the above-mentioned steps), “partially compliant” (1–5 steps), or “non-compliant” (0 steps). CHWs measured the mid upper arm circumference (MUAC) of the enrolled mothers during enrollment visits. Data on antenatal complications (except fever) were self-reported by respondent women. CHWs measured axillary temperature from women who reported having fever during the interview.

### Assessment of outcome variable

The primary outcome was preterm birth as defined by the World Health Organization as: “Any birth before 37 completed weeks of gestation or fewer than 259 days since the first day of the women’s LMP” [14]. Gestational age at birth was computed from the difference between the date of pregnancy outcome and the date of the first day of the LMP recorded at enrolment. Date of first day of the LMP was determined through maternal report to a CHW during a two-monthly pregnancy surveillance visit at the household, when the CHW asked the pregnant women to recall LMP with the assistance of calendars and memory aids. Women for whom no date of LMP was estimated after several attempts using various approaches were excluded from analysis. Date and type of pregnancy outcome was recorded by a CHW on her first visit after women delivered, usually within 24 hours or as soon as possible after birth.

### Data quality assurance

CHWs received 6 weeks of classroom-based and hands-on supervised training. These training sessions followed a structured curriculum including skills development for behavior change communication, provision of BNCP and essential newborn care, clinical assessment of neonates, and identification and management of sick newborns using an algorithm adapted from Integrated Management of Childhood Illness. Quality of data collected by CHWs was ensured through direct supervision by respective Field Supervisors. Supervisory visits and standardization exercise sessions were organized routinely to ensure quality of data collected. Every reported neonatal death was confirmed by a repeat visit to the household by a supervisory staff. A sample (5%) of newborns who survived the neonatal period was revisited for quality assurance of vital status reporting by CHWs.

CHWs submitted data forms to their supervisor, who checked the forms for completeness and consistency. Data entry system was custom-designed with built in range and consistency checks. Field verifications were conducted to resolve identified inconsistencies and incompleteness if required.

### Statistical analyses

Births at ≥37 weeks were classified as term births. Preterm births were sub-categorized as: 1) Very preterm (28 – 31 weeks of gestation), 2) Moderate preterm (32–34 weeks of gestation) and 3) Late Preterm (35–36 weeks of gestation). We estimated the incidence of preterm birth by dividing all live preterm births, whether singleton, twin or higher order multiples, by all live births in the population; 95% confidence intervals (CI) were calculated for the estimated proportion of preterm birth incidence.

Based on published reports and considering biological plausibility, we categorized, *a priori,* the potential risk factors for preterm birth into three groups: 1) Proximal factors: antenatal care seeking and antenatal complications during the index pregnancy; 2) Intermediate factors: previous pregnancy history; 3) Distal factors: socio-demographic characteristics. We constructed a wealth index score [15] for each household by employing principal component analysis of basic housing construction materials (i.e., materials used to construct the walls, roof, and floor of houses), sources of water, sanitation facilities and household belongings.

Covariates showing moderate strength of association (*P* <0.10) in bivariate analysis were included in multivariate models, which were constructed in a three-step sequence starting with proximal factors [Model1] and progressively adding previous pregnancy/birth information [Model2] and thereafter distal/socio-demographic factors) [Model3]. The association (risk ratio) between potential risk factors and preterm birth was modeled using binomial regression with a log link function; generalized estimating equations with exchangeable correlation structure were used to adjust standard errors to account for clustering [16,17]. In case of convergence failure, poisson models with robust standard error estimation were used [18,19]. Imputation of missing data was done using the “hotdeck” method by cluster [20]. Data were analyzed by using STATA (version 11) statistical package [21].

### Ethical approval

We received ethical approval for the study from the Institutional Review Board of Johns Hopkins Bloomberg School of Public Health and from the Ethical Review Committee of International Centre for Diarrheal Disease Research, Bangladesh (icddr,b). The study was registered at ClinicalTrials.gov (NCT00434408).

## Results

**Figure 1:**
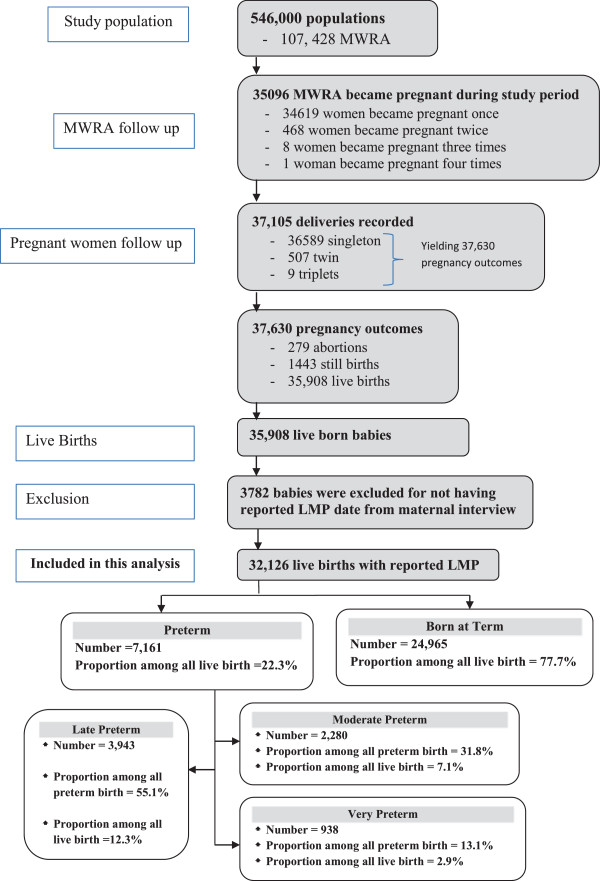
Study profile.

A total of 37,630 pregnancy outcomes, including 35,908 live births (Figure 1: Study Profile), were recorded in this study. Most women (89%) were able to recall the date of the LMP when facilitated by a CHW using Bangla calendar dates and reference to important social events. Excluding 3,782 women who could not report their LMP date, a total of 32,126 live births were included in this analysis. More than one-fifth [22.3%; 95% CI: 21.5, 23.1] of the live births were born prior to 37 weeks gestation. The proportions of late, moderate and very preterm births were 12.3% (95% CI: 11.8, 12.8), 7.1% (95% CI: 6.7, 7.5) and 2.9% (95% CI: 2.7, 3.2), respectively (Figure 1).

### Socioeconomic and background characteristics: distal factors

The majority (61.7%) of respondents was in the 20–29 year age group and most of them (95.5%) were Muslim (Table 1). More than half (51.9%) of the respondent, and 43.9% of their husbands had primary or higher level of education. Higher education level [5 or more years of schooling (Primary plus)] of both the mother [Relative Risk (RR): 0.92; 95% CI: 0.88, 0.97] and her husband (RR: 0.94; 95% CI: 0.89, 0.99) was associated with lower risk of preterm birth compared to lower education level. Compared to the highest wealth quintile group, the lowest quintile group of respondents had a 37.0% higher risk of preterm delivery (RR: 1.37; 95% CI: 1.26, 1.49).

**Table 1:** Association of selected background characteristics with preterm birth

**Characteristics**	**Live births (n = 32,126)**	**Preterm births (n = 7,161)**		**RR**	**95% CI**	**P value**
	**Number**	**Number**	**Percent**			
Women’s age at delivery						
<20 years	1874	391	20.9	0.84	0.75-0.93	<0.01
20-24 years	8993	1831	20.4	0.81	0.76-0.87	<0.001
25-29 years	10826	2412	22.3	0.89	0.84-0.95	<0.001
30-34 years	6479	1539	23.8	0.95	0.89-1.02	>0.15
≥35 years	3954	988	25.0	Ref.	-	
Women’s education^*^						
Below primary	15460	4009	25.9	Ref.	-	
Primary plus	16666	3152	18.9	0.73	0.70-0.76	<0.001
Husbands’ education^*^						
Below primary	18025	4522	25.1	Ref.	-	
Primary plus	14101	2639	18.7	0.75	0.71-0.78	<0.001
Religion						
Muslim	30684	6851	22.3	Ref.	-	
Others	1442	310	21.5	0.96	0.87-1.06	>0.45
Wealth quintile (Asset index score)						
Lowest quintile (Poorest)	6283	1667	26.5	1.86	1.73-2.00	<0.001
Second lowest	6475	1691	26.1	1.83	1.71-1.97	<0.001
Middle quintile	6332	1541	24.3	1.71	1.59-1.84	<0.001
Second highest	6442	1323	20.5	1.44	1.34-1.56	<0.001
Highest quintile (Richest)	6594	939	14.2	Ref.	-	

^*^Level of Education: Below primary = 0–4 years of schooling; Primary Plus = ≥5 years of schooling.

### Pregnancy related data: intermediate factors

The index pregnancy was fourth or higher order for 42.4% of respondent women (Table 2). Compared to women having fourth or higher birth order, primigravid women had 9.0% lower risk of delivering a preterm baby (RR: 0.91; 95% CI: 0.84, 0.98). From 18.2% of women, a history of child death was reported, which was associated with higher risk of preterm birth (RR: 1.05; 95% CI: 1.01, 1.10). A woman who delivered twins or triplets was at about 1.6-fold higher risk of preterm delivery (RR: 1.61; 95% CI: 1.49, 1.74) compared to a woman who delivered a singleton baby. Among all live births reported, 52.0% were boys; a female baby was 9.0% less likely to be preterm than a male baby (RR: 0.91; 95% CI: 0.88, 0.95).

**Table 2:** Association of women’s obstetric history and pregnancy outcome with preterm delivery

**Obstetric history**	**Live births (n = 32,126)**	**Preterm births (n = 7,161)**		**RR**	**95% CI**	**P value**
	**Number**	**Number**	**Percent (row)**			
Birth Order						
First child	6886	1343	19.5	0.80	0.76 – 0.85	<0.001
Second child	6290	1371	21.8	0.90	0.85 – 0.95	<0.001
Third child	5315	1135	21.4	0.88	0.83 – 0.93	<0.001
Fourth or higher	13635	3312	24.3	Ref.	-	
Sex of the baby delivered						
Female	15414	3277	21.3	0.91	0.88 – 0.95	
Male	16712	3884	23.2	Ref.	-	
Multiple pregnancy						
Yes	835	367	44.0	2.02	1.87 – 2.19	<0.001
No	31291	6794	21.7	Ref.	-	
History of still birth or abortion						
Yes	7898	2014	25.5	0.99	0.94 – 1.05	>0.73
No	24228	5177	21.4	Ref.	-	
History of child death						
Yes	5853	1295	22.1	1.20	1.15 – 1.25	<0.001
No	26273	5866	22.3	Ref.	-	

### Antenatal health, complication and antenatal care: proximal factors

Most women (57.3%) sought antenatal care at least once, 79.5% reported receiving any dose of tetanus toxoid (TT) immunization, and 89.9% consumed iron-folic acid (IFA) during the index pregnancy (Table 3). About one third (32.0%) of women practiced all six steps for BNCP while another 58.6% practiced partially. Antenatal complications of pregnancy were reported by 1.6% of women.

**Table 3:** Association of women’s antenatal care status and risk of preterm birth

**Characteristics**	**Live births (n = 32,126)**	**Preterm births (n = 7,161)**		**RR**	**95% CI**	**P value**
	**Number**	**Number**	**Percent (row)**			
ANC visits						
No ANC visit	13732	3568	26.0	Ref.	-	
At least one ANC	18394	3593	19.5	0.75	0.72 – 0.78	<0.001
Iron (tablet) consumption						
No Iron consumed	3231	646	20.0	Ref.	-	
Consumed for less than 60 days	4307	1007	23.4	1.17	1.07 – 1.28	<0.001
Consumed for 60 – 180 days	23520	5307	22.6	1.12	1.05 – 1.21	<0.005
Consumed for 181 days or more	1068	201	18.8	0.94	0.82 – 1.08	>0.40
Birth preparedness status						
Didn’t practice at all	3011	1799	59.7	Ref.	-	
Practiced partially	18838	3509	18.6	0.31	0.30 – 0.32	<0.001
Practiced fully	10277	1853	18.0	0.30	0.29 – 0.32	<0.001
Any dose of TT immunization during pregnancy						
Yes	25536	5562	21.8	0.90	0.86 – 0.94	<0.001
No	6590	1599	24.3	Ref.	-	
Mid-upper arm circumference (MUAC)						
<214 mm	6068	1580	26.0	1.56	1.45 – 1.67	<0.001
214 – 221 mm	5311	1309	24.6	1.47	1.37 – 1.59	<0.001
222 – 250 mm	15072	3324	22.1	1.32	1.24 – 1.41	<0.001
> = 251 mm	5675	948	16.7	Ref.	-	
Any reported antenatal complication^*^						
Yes	499	129	25.9	1.16	1.02 – 1.35	<0.05
No	31627	7032	22.2	Ref.	-	

^*^History of fever (or as measured by CHW), vaginal bleeding, swelling of hand, leg or face, severe abdominal pain, convulsion, severe headache, blurring of vision during pregnancy.

Bivariate association of each of these proximal factors with preterm birth risk is presented in Table 3. Compared to no visit at all, any ANC visit was associated with 25.0% lower risk of preterm birth (RR: 0.75; 95% CI: 0.72, 0.78). Risk of preterm delivery was also lower for women who undertook all BNCP steps (RR: 0.30; 95% CI: 0.29, 0.32) and women who received any dose of TT vaccine during pregnancy (RR: 0.90; 95% CI: 0.86, 0.94). Almost one-fifth (18.9%) of the respondents had a MUAC <214 mm. Maternal MUAC was inversely associated with preterm birth risk; for each cm increase in average MUAC measures, risk of preterm birth was 5.6% lower (95% CI: 4.8% - 6.5%). Women who reported any antenatal complication had 16.0% higher risk of preterm delivery compared to women who did not report any antenatal complication (RR: 1.16; 95% CI: 1.02, 1.35). *Itemized risk analysis for each of the antenatal complications is presented as Additional file*1*: web-table S1 (available online).*

### Multivariable regression analysis

Risk estimates from bivariate analyses for proximal factors, specifically for any antenatal visit, any antenatal complication, birth preparedness steps, and any dose of TT immunization, were not changed significantly after adding all proximal factors into the multivariate Model-1 (Table 4). Association of preterm birth with low MUAC was attenuated while the risk associated with IFA consumption was increased in magnitude relative to the estimate in bivariate analysis. In model-2, intermediate factors were added to proximal factors and estimated risk of preterm birth was 8% higher (RR: 1.08; 95% CI: 1.03, 1.13) among women who had a previous child death. Women having lower birth order had lower risk of preterm birth. Also a female child appeared to have 7.0% lower risk (RR: 0.93; 95% CI: 0.90, 0.96) of being born preterm, compared to a male child. In model-3, distal factors were added; risk estimates were attenuated but remained significant for women with lower MUAC and women consuming IFA during pregnancy, relative to the first model. Longer periods of iron-folic acid intake were associated with higher risk of preterm birth (for less than 2 months RR: 1.32; 95% CI: 1.21, 1.45; for 2–6 months, RR: 1.33; 95% CI: 1.24, 1.44). Risk of preterm birth remained high, but was attenuated, for women who continued to receive IFA supplementation beyond 6 months (RR: 1.28; 95% CI: 1.11, 1.47). Additionally, women who had a previous child death, had multiple pregnancy, had any antenatal complication and were in the poorest quintile were at significantly higher risk of preterm delivery, while at least one ANC visit, primigravida, adoption of all 6 steps for BNCP, education at primary or higher level, and TT immunization provided protection.

**Table 4:** Risk factor analysis for preterm birth using multivariable model^**^

**Risk factors**	**Model 1: proximal factors**		**Model 2: intermediate factors added**		**Full (final) model: distal factors added**	
	**RR**	**95% CI**	**RR**	**95% CI**	**RR**	**95% CI**
At least one ANC visit (Ref.: “No visit”)	0.79	0.75-0.82	0.80	0.77-0.84	0.86	0.83 – 0.90
Any antenatal complication (Ref.: “No complication”)	1.31	1.13-1.53	1.31	1.13-1.52	1.32	1.14 - 1.53
Birth preparedness Status						
No step was taken	Ref.	-	Ref.	-	Ref.	-
Partially	0.30	0.29-0.31	0.30	0.29-0.32	0.30	0.29 – 0.31
Fully	0.30	0.29-0.32	0.31	0.29-0.33	0.32	0.30 – 0.34
Iron tablet consumption						
No iron consumed at all	Ref.	-	Ref.	-	Ref.	-
Consumed for less than 60 days	1.43	1.31-1.56	1.39	1.27-1.52	1.32	1.21 – 1.45
Consumed for 60 – 180 days	1.43	1.32-1.55	1.39	1.29-1.50	1.33	1.24 – 1.44
Consumed for 181 days or more	1.34	1.16-1.55	1.32	1.15-1.53	1.28	1.11 – 1.47
Any dose of TT immunization (Ref: “Received no TT”)	0.94	0.90-0.99	0.94	0.89-0.98	0.95	0.91 – 0.99
Mid-upper arm circumference (MUAC)						
<214 mm	1.43	1.33-1.53	1.41	1.31-1.51	1.26	1.17 – 1.35
214 – 221 mm	1.37	1.27-1.47	1.36	1.27-1.46	1.23	1.14 – 1.33
222 – 250 mm	1.26	1.18-1.34	1.26	1.18-1.40	1.17	1.10 – 1.24
> = 251 mm	Ref.	-	Ref	-	Ref	-
Multiple pregnancy (Ref.: “Singleton”)			1.61	1.49-1.74	1.61	1.49 – 1.74
Birth Order						
First child			0.82	0.77-0.87	0.91	0.84 – 0.98
Second child			0.90	0.85-0.95	0.98	0.92 – 1.04
Third child			0.89	0.84-0.95	0.95	0.89 – 1.01
Fourth or higher			Ref.	-	Ref.	-
Sex of the baby delivered (Ref: Male child)			0.93	0.90 – 0.96	0.92	0.89 – 0.96
History of child death (Ref.: “No child death in past”)			1.08	1.03-1.13	1.05	1.01 – 1.10
Women’s age at delivery						
<20 years					0.97	0.87 – 1.09
20-24 years					0.93	0.86 – 1.01
25-29 years					0.95	0.89 – 1.02
30-34 years					0.99	0.92 – 1.05
35 years & above					Ref.	-
Women’s education: Primary or above (Ref.: “Below primary”)					0.92	0.88 – 0.97
Husbands’ education: Primary or above (Ref.: “Below primary”)					0.94	0.89 – 0.99
Wealth Index						
Lowest quintile (Poorest)					1.37	1.26 – 1.49
Second lowest quintile					1.47	1.35 – 1.59
Middle quintile					1.43	1.33 – 1.54
Second highest quintile					1.31	1.22 – 1.42
Highest quintile (Richest)					Ref.	-

^**^Estimates account for the cluster-randomized design of the study.

## Discussion

We have presented the incidence of and risk factors for preterm birth using prospectively collected data from a large cohort of 32,126 pregnant women in a rural population of Bangladesh who delivered a live-born infant. Among live-born babies, more than one-fifth was preterm (22.3%) and the majority of preterm births (55.1%) were late preterm. If we could support these pregnancies to continue an additional 1–2 weeks, that could lead to a substantial decrease in the preterm birth toll and burden of disease due to preterm. A small of number of behavioral (e,g. smoking cessation), clinical (e,g. progesterone supplementation) and health system interventions (e,g. reducing non-medically indicated labor induction or caesarean delivery) have been shown to reduce the preterm birth rate [3,22]. Bangladesh was ranked 7^th^ on the top-10 country list for high preterm birth rates in 2010 [1]. Although recent global estimates reported that sub-Saharan Africa and South Asia account for the majority (60%) of the globally estimated 14.9 million annual preterm births [1], available data on preterm birth rates from South Asian countries are scarce. Our estimate of 22.3% is consistent with data from similar regional community-based research sites in southern Nepal (NNIPS, Sarlahi, Nepal - 19%, [23,24]) and northwestern Bangladesh (JiVITA, Gaibandha, Bangladesh – 23%) [25].

Maternal factors increasing the risk of preterm birth in our population included socioeconomically poorer status, poor nutritional status (lower MUAC), antenatal iron consumption, history of a previous child death, multiple pregnancies and having any antenatal complication. Factors which were protective for preterm birth included practicing all 6 BNCP steps, first child, education at primary level (grade 5) or above, TT immunization, and female sex of the baby. Study results from Ahmedabad, India [26] reported previous child death as a risk factor for preterm birth, ranging from 1.5 times (1 child death; 95% CI: 0.9, 2.2) to 3.1 times (2 child deaths; 95% CI: 1.5, 6.4), compared to those who had no previous child death. Unlike our study, they found that first pregnancy was a risk factor as well (RR: 1.3; 95% CI: 0.9 – 1.9). Multiple gestations—accounting for only 2–3% of infants—carry a substantial risk of preterm delivery, resulting in 15–20% of all preterm births; nearly 60% of twins are born preterm [27]. A Zimbabwe study [28] reported similar increased risk of preterm birth attributable to multiple gestations (RR: 3.45; 95% CI: 3.1, 3.8) as ours.

Maternal nutritional status before and during pregnancy may contribute to the risk for preterm birth [29]. In the Preterm Prediction Study, a low pre-pregnancy body mass index (BMI) was strongly associated with increased risk of preterm birth, with the RRs being greater than 2.5 [30]. In contrast, a recent meta-analysis found that pre-pregnancy BMI had little or no relationship with the risk of preterm birth overall [31]. In our study, we found women having lower MUAC were at higher risk of preterm birth. Preterm birth can be caused by maternal thinness associated with decreased blood volume and reduced uterine blood flow [32]. Further research is required to understand the relationship between BMI and preterm birth risk.

Our finding on consumption of IFA during pregnancy as a risk factor for preterm birth differs from conventional knowledge and previous study reports. Traditionally, gestational anemia has been prevented with the provision of daily iron supplementation throughout pregnancy [33]. Results from a recent systematic review [34] which included 48 randomized trials and 44 cohort studies, revealed significant effect of prenatal iron consumption on reducing risk of low birth weight (RR: 0.81; 95% CI: 0.71, 0.93) but non-significant effect on preterm births (RR: 0.84; 95% CI: 0.68, 1.03).

However, many studies also fail to show beneficial effects of antenatal iron supplementation on pregnancy outcomes. Cochrane review and meta-analyses [35-37] concluded that iron supplementation during pregnancy was neither beneficial nor harmful in terms of preterm birth [38]. There is evidence to suggest that increasing iron intake is not always beneficial [39]; iron availability influences the severity and chronicity of maternal infections and thus might lead to negative pregnancy outcome, including preterm birth [40]. Because iron allays the fall in hemoglobin during pregnancy, iron-induced macrocytosis could increase blood viscosity to a degree that would impair utero-placental blood flow, decrease placental perfusion and increase risk of placental infarction [41,42]. This patho-physiological pathway may partially explain the results found in our study.

Education is the dimension of socioeconomic status that most strongly and consistently predicts health status [43,44]. A low level of education limits a person's access to employment and other social resources, which in turn limits his/her capacity to integrate within society and thereby increases the risk of subsequent poverty [43-45]. Similar to ours, studies in India and Brazil [26,46] also reported maternal education below primary level as a risk factor for preterm birth (RR: 1.4; 95% CI: 1.1, 1.8). We found that male sex was associated with preterm birth relative to girls, which is consistent with previous studies in which 55% of all preterm births are boys [47,48].

Studies from several developing countries have found that “no ANC visit” is a significant risk factor for preterm birth, ranging from 1.3 times to 7 times higher than for women having any ANC visit [26,28,46,49,50]. Visits to ANC centers and/or receiving ANC may raise awareness of the need for skilled delivery care [51] or give women and their families familiarity with the health services available at health centers or the skills of the service providers, thus enabling them to navigate and receive necessary care when a crises arises [52].

The main strengths of this study are that we analyzed prospectively collected, population-based data from a large sample (n = 32,126) of live births. In addition, since we collected data through visiting study women at home, the common concerns about selection bias in hospital-based studies from developing countries were avoided.

A limitation of our study was reliance on LMP to determine gestational age. Some of the common criticisms of this method are possible inaccurate recall of LMP, including heaping on certain dates, and reliance of the calculations on a “normal” menstrual cycle of 28 days with ovulation on day 14 [53-55]. However, mounting evidence shows that LMP dates can be utilized to estimate gestational age accurately and reliably [56] with a precision of 86%-90% [57]. In a multi-country trial in developing countries that enrolled 799 women from China, Cuba, and India, 99.9% of women were able to provide an exact date for their LMP, and 92.4% of LMP dates were within 1 week of physician clinical assessments [58]. In a study of 355 preterm neonates born in Dhaka Shishu Hospital, LMP estimates of gestational age were on average only 1 day lower than first or second trimester ultrasound determined gestational age (+/- 11 days) [57]. Compared to ultrasound, use of LMP may over or underestimate preterm delivery depending on characteristics of the sample, timing of ultrasound, and LMP recall period [53,57,59,60]. Such misclassification of gestational age estimation using the LMP method, along with differential misclassification across the risk factors of interest, could lead to over or underestimation of the population level burden of preterm birth.

Since preterm birth is the leading cause of neonatal deaths globally [61], and the second leading cause of deaths in children under five years of age, progress towards achieving MDG4 for child survival requires achieving higher coverage of evidence-based interventions to prevent preterm birth and/or to improve survival for preterm newborns [62]. Global experts announced a “Goal for reduction of preterm birth rate by 2025” on World Prematurity Day (Nov 11) in 2012 [3]. For countries, like Bangladesh, with a neonatal mortality rate above 5 per 1000 live births in 2010, “the goal is to reduce their preterm birth-attributable mortality by 50% between 2010 and 2025” [3]. Thus, it is important to ensure effective planning and design of community-based programs focusing on preterm births, specifically in low resource settings. Such a focus will require a clearer understanding of associated risk factors, especially those which can be intervened upon.

## Conclusion

In addition to demonstrating high burden of preterm births in a rural area in Bangladesh, our study has identified several risk factors for preterm birth, and thus can inform resource planning and design of community-based interventions to reduce mortality from preterm birth. Recognizing that the majority of preterm births are late or moderate preterm (during 32–36 weeks gestational age), even small reductions in the rates of these categories of preterm birth would mean sizable decreases in the number of overall preterm deliveries. From a program planning perspective, it is important to take risk factors for preterm birth into account, since addressing women at higher risk could help reduce late and moderate preterm births. Focusing on factors shown to reduce risk for preterm births (e, g. ANC visit, BNCP, TT immunization, surveillance for maternal complication during pregnancy) also can help community-based programs to reduce the preterm birth toll at population level. Our finding that preterm birth was associated with IFA consumption during pregnancy reveals one critical area for future studies. Better understanding of the relationship between maternal nutritional status and risk of preterm birth is needed. Overall, given the range of variability of preterm birth risks among ethnic and socioeconomic groups, similar studies should be conducted to generate more population-based evidence on burden and risk factors of preterm birth in developing countries.

## Abbreviations

ANC: Antenatal care; BMI: Body mass index; BNCP: Birth and newborn care preparedness; CDK: Clean delivery kit; CHW: Community Health Worker; IFA: Iron-folic acid; LMP: Last menstrual period; MUAC: Mid-upper arm circumference; ProjAHNMo: Project for Advancing Health of Newborns and Mothers; TT: Tetanus toxoid.

## Competing interests

The authors declare that they have no competing interests.

## Authors’ contributions

RS, AHB and LCM were primarily responsible for conceptualizing and designing this study. LCM, AHB, SEA and GLD were responsible for protocol development and study design of the main study (Chlorhexidine trial). AHB and SEA were the principal investigators of the ProjAHNMo Chlorhexidine study. RS, IM, SMR, GLD, LCM were co-investigators of the main study. RS conducted data analyses and drafted the manuscript. RRT, JAA, DM and NB contributed in literature review, interpretation of results and manuscript editing. DM and NB assisted in data management and analyses. All authors provided critical intellectual input in editing and revising the manuscript and approved the manuscript for submission.

## Pre-publication history

The pre-publication history for this paper can be accessed here:

http://www.biomedcentral.com/1471-2431/14/112/prepub

## Supplementary Material

Additional file 1: Web-Table S1 Reported antenatal complications and risk of preterm birth. Click here for file
