# Supplementary material for: Incidence and risk factors of preterm birth in a rural Bangladeshi cohort
Source: BMC Pediatr. 2014 Apr 24;14:112. doi: 10.1186/1471-2431-14-112 (PMC4021459; doi:10.1186/1471-2431-14-112)
Supplement: Additional file 1: Web-Table S1 — Reported antenatal complications and risk of preterm birth. [file 1471-2431-14-112-S1.docx]

**Additional file 1. Web-Table: Reported antenatal complications and risk of preterm birth**

| Reported complications during pregnancy | Live births (n=32,126) | Preterm births (n=7,161) | | RR | 95% CI | | P value | |
| --- | --- | --- | --- | --- | --- | --- | --- | --- |
|  | Number | Number | Per cent  (Row) |  |  | |  | |
| Fever | | | | | | | | |
| Yes | 133 | 25 | 18.8 | 0.84 | | 0.59 – 1.20 | | 0.34 |
| No | 31,993 | 7,136 | 22.3 | Ref. | |  | |  |
| Swelling of hand, leg or face | | | | | | | | |
| Yes | 150 | 31 | 20.7 | 0.93 | | 0.68 – 1.27 | | 0.64 |
| No | 31,976 | 7,130 | 22.3 | Ref. | |  | |  |
| Vaginal bleeding | | | | | | | | |
| Yes | 92 | 24 | 26.1 | 1.17 | | 0.83 – 1.65 | | 0.37 |
| No | 32,034 | 7,137 | 22.3 | Ref. | |  | |  |
| Convulsion | | | | | | | | |
| Yes | 46 | 7 | 15.2 | 0.68 | | 0.34 – 1.35 | | 0.27 |
| No | 32,080 | 7,154 | 22.3 | Ref. | |  | |  |
| Severe headache | | | | | | | | |
| Yes | 88 | 21 | 23.9 | 1.07 | | 0.74 – 1.56 | | 0.72 |
| No | 32,038 | 7,140 | 22.3 | Ref. | |  | |  |
| Blurring of vision | | | | | | | | |
| Yes | 107 | 26 | 24.3 | 1.09 | | 0.78 – 1.52 | | 0.61 |
| No | 32,019 | 7,135 | 22.3 | Ref. | |  | |  |
| Severe abdominal pain | | | | | | | | |
| Yes | 129 | 31 | 24.0 | 1.08 | | 0.79 – 1.47 | | 0.63 |
| No | 31,997 | 7,130 | 22.3 | Ref. | |  | |  |
